# Functional and structural impairments of the pulmonary system in middle-aged people with cervical and upper thoracic spinal cord injuries

**DOI:** 10.1080/10790268.2022.2031478

**Published:** 2022-02-23

**Authors:** Mattias Hill, Sophie Jörgensen, Gunnar Engström, Margaretha Persson, Per Wollmer, Jan Lexell

**Affiliations:** 1 Department of Health Sciences, Lund University, Lund, Sweden; 2 Department of Rehabilitation Medicine, Skåne University Hospital, Lund, Sweden; 3 Department of Clinical Sciences in Malmö, Clinical Research Centre, Lund University, Malmö, Sweden; 4 Department of Internal Medicine, Skåne University Hospital, Malmö, Sweden; 5 Department of Translational Medicine, Lund University, Malmö, Sweden

**Keywords:** Lung diseases, Respiratory function tests, Respiratory insufficiency, Spinal cord injuries

## Abstract

**Objectives:**

To describe functional and structural impairments of the pulmonary system in middle-aged people with cervical and upper thoracic spinal cord injuries (SCI), and compare findings to the general population. To determine if the neurological level of injury (NLI) is related to functional and structural impairments, and if age is related to structural impairments.

**Design:**

Cross-sectional study with matched controls. Data from the Swedish SPinal Cord Injury Study on Cardiopulmonary and Autonomic Impairment (SPICA). Matched control data were obtained from the Swedish CArdioPulmonary bioImage Study (SCAPIS).

**Setting:**

Outpatient SCI unit in southern Sweden.

**Participants:**

25 people (20% women, mean age 58 years, mean time since injury 28 years, NLIs C2-T6, American Spinal Injury Association Impairment Scale A-C).

**Interventions:**

Not applicable.

**Outcome measures:**

Lung function was assessed with spirometry, diffusing capacity and impulse oscillometry. Structural assessments were performed with computed tomography.

**Results:**

Pulmonary function was generally worse compared to the controls. Structural impairments were common (75% of the participants and 65% of the controls; P = 0.36, NS). NLI was significantly related to some of the functional and structural impairments.

**Conclusions:**

Middle-aged people with long-term cervical and upper thoracic SCI can have substantial pulmonary functional impairments, whereas structural impairments do not differ considerably from the general population. Further larger and longitudinal studies should focus on the clinical impact of these impairments over time.

## Introduction

Cervical and upper thoracic spinal cord injury (SCI) leads to paralysis of the respiratory muscles, affecting both inspiratory and expiratory function.^1^ This, in turn, causes a restrictive pulmonary disease with reduction in total lung capacity.^1^ Expiratory muscle weakness impairs effective coughing and thereby, the risk for retention of secretions and lower respiratory tract infections increases.^1^ Consequently, respiratory diseases are the leading cause of death in people with SCI together with cardiovascular disease (CVD), where the neurological level of injury (NLI) and severity of injury are the most important predictors of pulmonary dysfunction and mortality.^1,2^

To advance the clinical management, there is a need for detailed knowledge about long-term pulmonary functional and structural impairments in people with a cervical or upper thoracic SCI. Previous research on pulmonary dysfunction in SCI has mainly been performed in the early stages after SCI,^3–7^ and comparisons with matched controls from the general population are lacking.

To contribute with new knowledge in this field, we initiated the Swedish SPinal Cord Injury Study on Cardiopulmonary and Autonomic Impairment (SPICA).^8^ SPICA is a broad and in-depth assessment of the cardiopulmonary and autonomic systems in a cohort of middle-aged people with cervical and upper thoracic SCI. In the first study, the methodology, cohort demographics and initial results of SPICA have been described in detail.^8^

The objectives of the present study are: (1) to describe functional and structural impairments of the pulmonary system in middle-aged people with cervical and upper thoracic SCI and to determine if findings are different from the general population, and (2) to determine if NLI is related to functional and structural impairments, and if age among the participants and controls is related to structural impairments. We hypothesize that functional and structural impairments of the pulmonary system in middle-aged people with cervical and upper thoracic SCI are more common than in the general population, and that NLI and age explain the variance in some of the functional and structural impairments.

## Methods

### Research design

This study is part of the Swedish SPinal Cord Injury Study on Cardiopulmonary and Autonomic Impairment (SPICA).^8^ SPICA is a cross-sectional cohort study targeting people with cervical and upper thoracic SCI and has a combined exploratory and descriptive research design. SPICA is registered at ClinicalTrials.gov (Identifier: NCT03515122; Registration date: 2 May 2018)*.* The study conforms to the STROBE (Strengthening the Reporting of Observational Studies in Epidemiology).^9^

SPICA is based on the baseline protocol of the Swedish CArdioPulmonary and bioImage Study (SCAPIS).^10^ SCAPIS, in turn, is a large Swedish multicentre study aiming to improve risk prediction and study disease mechanisms of CVD and chronic obstructive pulmonary disease (COPD) in a cohort of 30,000 people aged 50–64 years from the general population. Matched control data from SCAPIS were used to compare with data collected in SPICA.

### Ethics

SPICA was approved by the Regional Ethical Review Board in Lund, Sweden (No. 2017/756) and we followed the Declaration of Helsinki for research on humans.^8^ All participants signed a written informed consent form prior to enrollment. All participants received written and oral information of their study findings at the end of the study. Pathological findings were managed to establish further investigations or treatment.

### The SPICA study population

The inclusion criteria of SPICA were: (i) traumatic SCI, American Spinal Injury Association Impairment Scale (AIS)^11^ A-C, NLI C1-T6; (ii) age 50–65 years; (iii) time since injury five years or more; (iv) resident in the Skåne region, southern Sweden. People who were ventilator dependent were excluded, due to the inability to participate in several of the assessments.

The participants were recruited from the SCI Unit, Department of Rehabilitation Medicine, Skåne University Hospital in Lund, Sweden.^8^ The SCI unit is responsible for the lifelong follow-up of all people with SCI in the Skåne region, Sweden (total population of about 1.3 million people).

A total of 38 people met the inclusion criteria and the final sample comprised 25 participants (response rate 66%). No power calculation was made due to the exploratory and descriptive design of SPICA. There were no significant differences between the 25 participants and the 13 non-participants regarding age, sex and injury characteristics (NLI, AIS grade, age at injury and time since injury). Therefore, we have concluded previously that our sample is likely to represent the population of middle-aged people with cervical and upper thoracic SCI in the Skåne region, Sweden.^8^

### Data collection procedure in SPICA

The data collection for the 25 participants in SPICA was performed on three separate occasions at the SCAPIS study site at Skåne University Hospital in Malmö, Sweden, during the SCAPIS’ data collection and the time period January to May 2018. The assessments and included variables were the same as the SCAPIS’ baseline protocol. The first author took part in all data collection procedures. A subset of the collected data from SPICA^8^ was used to address the objectives of the present study. In Table 1, an overview and description of the assessments in the present study are presented.

**Table 1:** Functional and structural assessments of the pulmonary system.

Assessment	Definition of parameter
Functional assessments	
Spirometry	
Vital capacity (VC)	The volume change between full inspiration and complete expiration (L)
Slow vital capacity (SVC)	Vital capacity performed with an unforced and complete expiration (L)
Forced vital capacity (FVC)	Vital capacity performed with a maximally forced and complete inspiration/expiration (L)
Forced expiratory volume in 1 s (FEV_1_)	The volume of air exhaled during the first second of the FVC (L)
FEV_1_/FVC	FEV_1_ to FVC ratio
MEF_50_	Maximal forced expiratory flow after 50% of the FVC has been exhaled (L/s)
Diffusing capacity	
Diffusing capacity of the lungs for carbon monoxide (D_LCO_)	Total CO uptake by the lung per unit of time per unit CO driving pressure (mmol/(min kPa))
Alveolar volume (V_A_)	Alveolar volume measured by gas dilution (L)
Carbon monoxide transfer coefficient (k_CO_)	The concentration fall in alveolar CO per unit CO driving pressure (ml/(min kPa L))
Impulse oscillometry	
Resistance at 5 Hz (R5)	The resistive component of the respiratory impedance including central and peripheral airways, lung tissue and chest wall resistance measured at 5 Hz (kPa/(L/s))
Resistance at 20 Hz (R20)	The resistive component of the respiratory impedance measured at 20 Hz, including mainly central airway resistance and to a lesser extent peripheral airways, lung tissue and chest wall resistance (kPa/(L/s))
Resistance at 5 Hz minus 20 Hz (R5-R20)	The resistive component of the respiratory impedance measured at 5 Hz minus the component measured at 20 Hz, indicating the resistive component of the small airways (kPa/(L/s))
Reactance at 5 Hz (X5)	The reactive component of the respiratory impedance incorporating the mass-inertive forces of the moving air column (inertance) and the elastic properties of lung periphery (elastance) at 5 Hz (kPa/(L/s))
Resonant frequency (Fres)	The point at which elastance and inertance are equal (Hz)
Reactance curve area below zero (AX)	The quantitative index of the total respiratory reactance between 5 Hz and the resonant frequency (kPa/L)
Structural assessments	
Computed tomography	
Emphysema	Focal/regional areas with low attenuation (Yes/No)
Bronchial wall thickening	Abnormal thickening of bronchial walls (Yes/No)
Bronchiectasis	Dilatation of bronchi with respect to the pulmonary artery, no tapering of bronchi and bronchi should be identified within 1 cm of the pleural surface (Yes/No)
Consolidation	Homogenous increase in the pulmonary parenchyma obscuring the margins of airway walls and vessels (Yes/No)
Cysts	A round area with well-defined interface with normal lung appearing lucent or low-attenuated (Yes/No)
Ground glass	An area of hazy increased opacity of lung. Bronchial and vascular margins are preserved (Yes/No)
Honeycombing	Cystic air spaces appearing in clusters with well-defined walls (Yes/No)
Linear scars of atelectasis	Reduced volume and increased attenuation with a subsegmental distribution and a linear configuration (Yes/No)
Mosaic attenuation	Regions appearing with a patchwork of differing attenuation (Yes/No)
Reticular abnormality	A collection of small linear opacities resembling a net constituted by interlobular septal thickening, intralobular lines or cyst walls of honeycombing (Yes/No)
Solid nodules	A rounded or irregular opacity with homogenous attenuation measuring up to 3 cm in diameter (Yes/No)
Lymphadenopathy	Enlarged mediastinal or hilar lymph node (Yes/No)
Any structural impairment	The presence of any of the abovementioned structural impairments (Yes/No)

CO, carbon monoxide; kPa, kilopascal.

#### Socio-demographics, medical history, injury characteristics, anthropometry and hemoglobin

A study-specific questionnaire was used to obtain data on age, sex, socio-demographic factors (marital status, educational level and vocational situation) and pulmonary history. Injury characteristics (age at injury, time since injury, AIS grade and NLI) were obtained from the medical records, interviews and clinical examinations. Body weight was recorded with a portable scale for wheelchairs (Corina Medical MPWS 300; Rörvik, Sävsjö, Sweden). Body height and waist circumference were measured in a supine position using a flexible measuring tape. Hemoglobin level was determined by routine analysis of a venous blood sample at the Department of Clinical Chemistry, Skåne University Hospital in Malmö, Sweden.

#### Functional assessments

All functional assessments (cf. Table 1) were performed with the participants seated upright in their wheelchair. Dynamic spirometry (Jaeger MasterScreen PFT, Carefusion, Hoechberg, Germany) was performed according to the American Thoracic Society (ATS)/European Respiratory Society standards (ERS), adjusted for SCI, to assess lung volumes and flows.^12,13^ The following spirometry parameters were measured: slow vital capacity (SVC), forced vital capacity (FVC), forced expiratory volume in 1 s (FEV_1_), FEV_1_/FVC and maximal expiratory flow at 50% (MEF_50_). These measures were used to calculate the predicted percent of vital capacity (VC), FEV_1_ and MEF_50_ according to established methods.^14^

Diffusing capacity (Jaeger MasterScreen PFT) was measured using the single-breath method according to ATS/ERS standards.^12^ The diffusing capacity of the lungs for carbon monoxide (D_LCO_), alveolar volume (V_A_) and the carbon monoxide transfer coefficient (k_CO_) were measured. These measures were corrected for hemoglobin levels and used to calculate the predicted percent of D_LCO_ and k_CO_.^15,16^

To assess respiratory impedance, impulse oscillometry (IOS)^17^ (Jaeger MasterScreen PFT) was performed to calculate R5, R20, R5-R20, X5, AX and resonant frequency (Fres). The SCAPIS participants at the Malmö study site performed the pulmonary function tests 15 min after the inhalation of 400 µg salbutamol, as the aim was to study COPD. For logistic reasons, this was not possible for the 25 participants in SPICA.

#### Structural assessments

High resolution computed tomography (CT; Siemens Definition Flash 2 × 128 slice, stellar detector, 4D-Care dose, Care-kV and SAFIRE, Forchheim, Germany) scans of the full lung volume were performed during inspiration and expiration to assess pulmonary structural impairment. The generated images were examined by an experienced thoracic radiologist to determine the presence (yes/no) of structural impairments according to international standards (cf. Table 1).^18^

### Matched control data from the general population

Matched control data at a ratio of 4:1 were obtained from the 6250 participants enrolled at the Malmö study site of SCAPIS from 2014 to 2018.^10^ All matching procedures and access to data were performed by SCAPIS after an application to the SCAPIS Data Mart (https://scapisdata.wlab.gu.se/Project/Index). The controls were matched for age (±1 year), sex, smoking history and height (±5 cm), as these variables are important determinants of pulmonary function.^12,19^ Weight or BMI were not used as matching variables as these are not considered comparable in people with SCI and the non-injured population, following body compositional changes after injury.^20^ The control data were collected during the same time period (six months) and by the same staff as in SPICA. If one participant had multiple available controls, they were chosen at random.

#### Statistical analysis

Data are presented using quantitative descriptive statistics with mean, median, standard deviation, minimum and maximum, and frequency and proportion (%), where appropriate.

Data from the participants and the matched data were compared using conditional logistic regression models. First, all data were assessed for normality. Two of the seventeen variables were highly skewed; therefore, AX (cf. Table 1) and pack years (cf. Table 2) were logarithmically transformed. If data from the 25 participants were missing, the corresponding control data were not included in the analyses. In a few cases, matched data were missing and sensitivity analyses with single imputation were performed.

**Table 2:** Socio-demographics, injury characteristics, pulmonary history, and anthropometry of the participants with spinal cord injury and the matched controls from the general population.^a^

	Participants with SCI	Matched controls	P value
Sex (n (%))			
Women	5 (20)	20 (20)	
Men	20 (80)	80 (80)	
Age (years) (mean ± SD; median, min-max)	58 ± 5; 58, 50–65	58 ± 4; 58, 50–64	0.74
Age at injury (years) (mean ± SD; median, min-max)	30 ± 12; 28, 7–56		
Time since injury (years) (mean ± SD; median, min-max)	28 ± 13; 31, 6–53		
Injury level (n (%))			
C1-C4	10 (40)		
C5-C8	6 (24)		
T1-T6	9 (36)		
Severity of injury (n (%))			
AIS A	15 (60)		
AIS B	8 (32)		
AIS C	2 (8)		
Socio-demographics (n %)			
Marital status^b^	18 (72)	77 (77)	0.77
Vocational situation			**<0**.**001**
Working full-time or part-time	10 (40)	79 (79)	
Disability pension, old age pension or paid sick leave	15 (60)	21 (21)	
Educational level			0.83
Low (Elementary school)	1 (4)	10 (10)	
Medium (Upper secondary school, vocational training)	14 (56)	47 (47)	
High (University or college degree)	10 (40)	43 (43)	
Smoking history			
Non-smoker (n (%))	11 (44)	44 (44)	
Ex-smoker (n (%))	12 (48)	48 (48)	
Current smoker (n (%))	2 (8)	8 (8)	
Pack years^c,d^ (mean ± SD; median, min-max)	8 ± 11; 4, 0–46	12 ± 20; 5, 0–140	0.18
Asthma (n (%))	2 (8)	4 (4)	
Chronic obstructive pulmonary disease (n (%))	0 (0)	0 (0)	
Anthropometrics (mean ± SD; median, min-max)			
Height^e^; cm	175 ± 8; 175, 160–190	175 ± 8; 175, 149–191	0.93
BMI; kg/m^2^	25.1 ± 5.3; 25.4, 14.1–34.4	27.4 ± 3.9; 26.9, 19.2–39.6	**0**.**015**
Waist circumference; cm	101 ± 14; 103, 70–127	97 ± 12; 96, 72–129	0.081

^a^ Comparison using conditional logistic regression analyses.

^b^ Living in a relationship (married/cohabiting/partner).

^c^ One pack years is defined as smoking 20 cigarettes (or equivalent consumption of cigar, pipe) each day for a whole year.

^d^ Conditional logistic regression computed on transformed data because of highly skewed data.

^e^The matching criteria for height had to be extended due to lack of controls in a few cases.

AIS, American Spinal Injury Association (ASIA) Impairment Scale^11^; BMI, body mass index; SCI, spinal cord injury.

The Spearman rank correlation coefficient (r_s_) was used to investigate associations between NLI (defined as each higher NLI C1-T6) and functional and structural impairments. Based on the descriptive results, the variables any structural impairment and linear scars of atelectasis (cf. Table 1) were included in the analyses.

Associations between chronological age and structural impairments were firstly investigated by calculating the odds ratios (OR) using logistic regression for the participants and the controls, respectively. Then, conditional logistic regression was used with age as an interaction variable (age multiplied with the presence of the structural finding) to investigate whether these ORs differed.

P values less than 0.05 were considered statistically significant. All statistical analyses were performed using the IBM SPSS Statistics Software v 25 (IBM Corporation, Armonk, NY, USA).

## Results

### Study population

In Table 2, the socio-demographics, medical history, injury characteristics, and anthropometry of the 25 participants with SCI and the matched controls (*n* = 100) from the general population are presented. The two groups had a comparable level of education (P = 0.83), whereas significantly fewer participants with SCI were working as compared to the controls (40% and 79%, respectively; P < 0.001). Eight percent in both groups were current smokers. Few participants and controls had asthma and none had COPD. None of the participants used a cough assist machine on a regular basis.^8^ The participants had a significantly lower BMI compared with the controls (25.1 and 27.4, respectively, P = 0.015).

### Descriptive findings of pulmonary functional and structural impairments, and comparison between the participants and the controls

In Table 3, the descriptive findings and the conditional regression analyses of the 25 participants and the controls are presented. The results of the functional assessments among the participants were generally low, expressed as % predicted, whereas the results among the controls were considered normal. In total, 18 participants (75%) and 62 controls (65%) had structural impairments; linear scars of atelectasis were the most common among the participants (n = 13; 54%).

**Table 3:** Descriptive findings and conditional logistic regression analyses of functional and structural impairments of the participants with SCI and matched controls from the general population.

	Participants with SCI (n = 25)	Matched controls (n = 100)	P value
Functional impairments^a^ (mean ± SD; median, min-max)			
Spirometry			
VC (% predicted)	69 ± 21; 70, 30–106	107 ± 14; 108, 63–150	**<0**.**001**
FEV_1_ (% predicted)	70 ± 24; 71, 33–111	109 ± 15; 108, 69–148	**<0**.**001**
FEV_1_/FVC	0.78 ± 0.10; 0.80, 0.48–0.93	0.80 ± 0.05; 0.80, 0.61-0.90	0.26
MEF_50_ (% predicted)	65 ± 30; 66, 17–115	100 ± 30; 97, 27–164	**<0**.**001**
Diffusing capacity			
D_LCO_ (% predicted)	64 ± 20; 65, 26–115	93 ± 12; 93, 63–117	**<0**.**001**
k_CO_ (% predicted)	90 ± 22; 84, 38–130	100 ± 14; 100, 71–129	**0**.**011**
Impulse oscillometry			
R5 (kPa/(L/s))	0.36 ± 0.11; 0.34, 0.19–0.66	0.30 ± 0.08; 0.29, 0.17–0.53	**0**.**018**
R20 (kPa/(L/s))	0.29 ± 0.08; 0.29, 0.16-0.54	0.26 ± 0.06; 0.25, 0.14–0.42	**0**.**038**
R5-R20 (kPa/(L/s))	0.062 ± 0.055; 0.050, −0.01–0.21	0.041 ± 0.043; 0.030, −0.03–0.26	0.087
X5 (kPa/(L/s))	−0.12 ± 0.07; −0.11, −0.32, −0.03	−0.08 ± 0.04; −0.07, −0.24, −0.03	**0**.**003**
Fres (Hz)	13.69 ± 4.99; 13.39, 7.42–24.65	10.60 ± 3.16; 9.85, 6.40–24.37	**0**.**002**
AX^b^ (kPa/(L)	0.55 ± 0.60; 0.39, 0.04–2.60	0.23 ± 0.28; 0.14, 0.02–2.05	**0**.**001**
Structural impairments^c^ (n (%))			
Emphysema	3 (13)	4 (4)	
Bronchial wall thickening	1 (4)	14 (15)	
Bronchiectasis	0 (0)	1 (1)	
Consolidation	1 (4)	1 (1)	
Cysts	1 (4)	2 (2)	
Ground glass	2 (8)	10 (10)	
Honeycombing	0 (0)	1 (1)	
Linear scars of atelectasis	13 (54)	35 (37)	
Mosaic attenuation	0 (0)	0 (0)	
Reticular abnormality	0 (0)	1 (1)	
Solid nodules	12 (50)	34 (35)	
Lymphadenopathy	1 (4)	1 (1)	
Any structural impairment	18 (75)	62 (65)	0.36

^a^In four participants, diffusing capacity could not be measured due to VC <1.5 L (n = 2) and logistical reasons (n = 2). IOS data were missing from two participants for logistical reasons.

^b^Conditional logistic regression computed on transformed data because of highly skewed data.

^c^Computed tomography assessments could not be performed in one participant for logistical reasons.

AX, Reactance curve area below zero; D_LCO_, diffusing capacity of lung for carbon monoxide; FEV_1 _, forced expiratory volume in 1 s; Fres, resonant frequency; FVC, forced vital capacity; k_CO_, carbon monoxide transfer coefficient; MEF_50_, maximal expiratory flow at 50%; R5, Resistance at 5 Hz; R5-R20, Resistance at 5 Hz minus 20 Hz; R20, Resistance at 20 Hz; SCI, spinal cord injury; VC, vital capacity; X5, Reactance at 5 Hz;

For each pulmonary function variable, there were significant differences between the participants with SCI and the controls, except for FEV_1_/FVC and R5-R20. No significant differences were found between the participants with SCI and the controls regarding the occurrence of structural impairments.

Control data from one person for forced spirometry were missing, from four people for the D_LCO_ and from one person for the IOS; the sensitivity analyses did not change any of the inferences.

### Associations between NLI and functional and structural impairments, and between age and structural impairments

In Table 4, associations between NLI and functional and structural impairments are presented. Higher NLI was significantly and negatively associated with each spirometry variable and positively associated with R5. Higher NLI was significantly and positively associated with the occurrence of any structural impairment (r_s _= 0.51; P *= *0.012).

**Table 4:** Spearman rank correlation between injury level and functional and structural impairments.

	Variable	Correlation coefficient (*r*)	P value
Injury level^a^	Functional impairments		
	VC (% predicted)	−0.58	**0**.**002**
	FEV_1_ (% predicted)	−0.57	**0**.**003**
	FEV_1_/FVC	−0.53	**0**.**007**
	MEF_50_ (% predicted)	−0.64	**0**.**001**
	D_LCO_ (% predicted)	−0.30	0.19
	k_CO_ (% predicted)	−0.02	0.94
	R5 (kPa/(L/s))	0.42	**0**.**046**
	R20 (kPa/(L/s))	0.36	0.090
	R5-R20 (kPa/(L/s))	0.26	0.24
	X5 (kPa/(L/s))	−0.40	0.058
	Fres (Hz)	0.34	0.12
	AX (kPa/(L)	0.33	0.12
	Structural impairments		
	Any structural impairment	0.51	**0**.**012**
	Linear scars of atelectasis	0.31	0.14

^a^ Defined as each higher Injury level C1-T6.

AX, Reactance curve area below zero; D_LCO_, diffusing capacity of lung for carbon monoxide; FEV_1_, forced expiratory volume in 1 s; Fres, resonant frequency; FVC, forced vital capacity; k_CO_, carbon monoxide transfer coefficient; MEF_50_, maximal expiratory flow at 50%; R5, Resistance at 5 Hz; R5-R20, Resistance at 5 Hz minus 20 Hz; R20, Resistance at 20 Hz; VC, vital capacity; X5, Reactance at 5 Hz.

In Table 5, associations between age and structural impairments are presented. Among the participants, age was significantly and positively associated with linear scars of atelectasis (OR = 1.3; 95% CI *= *1.04–1.74). There was no such association among the controls, and this difference was significant (P = 0.025).

**Table 5:** Associations between chronological age and structural impairments of the participants with SCI and matched controls, respectively, and comparison of ORs using conditional logistic regression analyses.^a^

	Participants with SCI (n = 24)	Matched controls (n = 96)	P value
Structural impairments (OR; 95% CI)			
Any structural impairment	1.04 (0.84–1.29)	1.03 (0.93–1.14)	0.88
Linear scars of atelectasis	1.3 (1.04–1.74)	0.9 (0.88–1.07)	**0**.**025**

^a^ Investigated using age as an interaction variable.

OR, odds ratio; SCI, spinal cord injury.

## Discussion

To improve the clinical management of pulmonary dysfunction in cervical and upper thoracic SCI, detailed assessments in people with long-term SCI are needed. In the present study, functional and structural impairments of the pulmonary system have been investigated and explored in detail in people with long-term cervical and upper thoracic SCI, and compared with matched data from the general population. In summary, the results of all functional assessments were generally worse among the participants compared to the matched controls. Structural impairments were common, but there were no significant differences between the participants and controls. The NLI was significantly related to some of the functional and structural impairments and age was significantly related to the presence of linear scars of atelectasis.

### Pulmonary functional impairments

Three different methods were used to provide a broad assessment of pulmonary function. As hypothesized, functional impairments were very common among the participants and pulmonary function was generally worse than among the controls.

Overall, the spirometry results among the participants were in agreement with studies of younger people with cervical and upper thoracic SCI,^3,21^ and significantly worse compared to the controls. All controls were assessed after treatment with salbutamol, which could have affected the magnitude of these differences. However, only four of the 100 controls were diagnosed with asthma and none with COPD (cf. Table 2). Therefore, we should not expect any major effects of salbutamol in the control group.

In relation to the predicted value, the D_LCO_ (64%) was lower than the k_CO_ (90%) among the participants. This is the result of the lower V_A_, which can be explained by the neuromuscular weakness, causing a lower total lung capacity and thereby a lower alveolar surface area. A study by Hart *et al*.^22^ including five participants with a combined inspiratory and expiratory neuromuscular weakness, had comparable results of 58% and 86%, respectively. In people with SCI, paradoxical movements of the rib cage occur during respiration^23^ causing an inhomogeneous distribution of aerated alveoli, which may further explain the lower diffusing capacity among our participants. Emphysema was also more common in the participants than the controls, which also contribute to the reduced D_LCO_ and k_CO_.^24^ Moreover, it is possible that altered cardiac function^25^ and reduced ventilation capacity^26^ affect the pulmonary vasculature. Further studies of the pulmonary vasculature may improve our understanding of diffusing capacity in cervical and upper thoracic SCI.

The diffusing capacity could not be analysed in two participants, as their VC were <1.5 L (cf. Table 3). This most likely led to a smaller difference between the participants and the controls, but we do not expect an impact on the overall inferences.

IOS assesses the mechanical properties of the pulmonary system. To our knowledge, IOS has not been studied in middle-aged people with SCI.^4–6^ Two components – resistance and reactance – are included in IOS (cf. Table 1). Our results indicate an increased airway resistance (R5 and R20; cf. Table 1) compared to controls, and the most plausible explanation is a reduced functional residual capacity in people with cervical and upper thoracic SCI.^1^ Lower functional residual capacity causes a collapse of alveoli and closure of small airways, which leads to an increase in airway resistance.^27^ In addition, interrupted sympathetic innervation has been hypothesized to cause a greater airway resistance in younger men with tetraplegia.^4,7^ The differences in airway resistance should be treated with some caution as salbutamol influenced the results of the controls. Salbutamol has been demonstrated to affect R20 more than the variability (decrease 8.3% at 200 µg).^28^ Thus, it is possible that the difference in R20 is overestimated and, accordingly, the difference in R5-R20 is underestimated.

The reactance includes the components inertance and elastance. Inertance refers to the inertia of the moving air column and elastance reflects the stiffness of the respiratory system.^17^ Our results demonstrated changes in elastance (X5, Fres and AX; cf. Table 1) which may be interpreted as increased stiffness or heterogeneity of the respiratory system compared to controls.^17^ The negatively affected elastance could be due to the limited ability to fully expand the chest wall to large lung volumes, which has been reported to decrease the elasticity of the lungs.^1,29^

Taken together, our results are in agreement with previous research of pulmonary dysfunction in younger people early after SCI. The functional assessments indicate a worse outcome of pulmonary complications when the cardiopulmonary demand increases. This is due to a reduced neuromuscular capacity, a lower diffusing capacity and mechanical properties of the pulmonary system causing a more strenuous respiration. These functional assessments contribute to a comprehensive understanding of pulmonary function, and may therefore be valuable tools in future evidence-based clinical routines.

### Pulmonary structural impairments

This is the first study to use CT to explore in detail pulmonary structural impairments in cervical and upper thoracic SCI. A higher NLI was related to a greater likelihood of any structural impairment suggesting that structural impairments are to some degree related to the SCI. An increased vulnerability of the respiratory tract and pulmonary functional impairments after SCI are possible explanations. However, we could not confirm our hypothesis that people with SCI have more structural impairments than the general population. This implies that structural impairments may also be related to ageing rather than the SCI.

The odds of having linear scars of atelectasis increased among the participants with a factor of 1.3 for each year of age, which was not the case among the controls. With increasing age, the elastic recoil of the lungs is reduced, which results in premature closure of peripheral airways.^30^ Thus, increasing age itself is a plausible explanation for the relationship between age and linear scars of atelectasis. In addition, the reduced neuromuscular capacity among the participants is likely to impair the opening of small airways during respiration, which possibly contributes to the difference compared to the controls.

We could also demonstrate that interstitial findings were rare among the participants, indicating that the long-term exposure to pulmonary dysfunction did not seem to affect the parenchyma. These results are new but not surprising as the pulmonary dysfunction in SCI is of extrapulmonary cause.^1^

Comparable studies are very rare, and we only found one study with a similar design. Estenne *et al*.^31^ used CT to assess microatelectasis in 14 people with respiratory muscle weakness, whereof eight with tetraplegia after SCI (seven younger, age 22–47 years, and one older, age 69 years). Our results are in agreement with this study, where only the older participant had microatelectasis.

From a clinical perspective, the association between linear scars of atelectasis and older age is noteworthy. The ageing-related loss of elastic recoil of the lungs leads to a risk for stagnation of respiratory secretion which contribute to increased susceptibility to pneumonia.^30^ Consequently, the incidence of people in the general population hospitalized with pneumonia in middle age is low, and then rapidly increases with advancing age.^32^ Interestingly, our knowledge of the incidence of pneumonia in people with cervical and upper thoracic SCI is limited.^33^ Among our participants, only one participant (age 62 years) had been hospitalized with pneumonia in the past five years.^8^ Further studies are needed to understand the impact of atelectasis among older people ageing with cervical and upper thoracic SCI and the risk for pneumonia. In future research, CT assessments of pulmonary structural impairments are considered valuable, and may subsequently be a tool in the clinical follow-up.

It is worth noting, though, that we only assessed the occurrence of structural impairments. We did not quantify or specify anatomical locations of the structural impairments. Our knowledge of pulmonary structural impairments in this population would be further enhanced with such an in-depth characterization.

Taken together, people ageing with a cervical and upper thoracic SCI may have an increased vulnerability to developing pulmonary structural impairments. Further larger and longitudinal studies are necessary to confirm this hypothesis.

## Strengths and limitations

A major strength of this study is the combination of several advanced assessments to provide an in-depth understanding of the pulmonary system, many that have not been used before in this population. Also, the recruitment of the participants was population-based and included women, and was considered representative of the population of middle-aged people with cervical and upper thoracic SCI in the Skåne region, Sweden. All assessments were performed over the same period of time, with the same equipment and by the same staff for both the participants with SCI and the controls. Moreover, the carefully matched control data from the general population allowed a detailed comparison with the SCI population, although the salbutamol exposure in the control group affects some of the results. The included assessments were not tailored for SCI, as we used the same protocol as SCAPIS. Important measures, for example, chest anthropometry and maximum inspiratory and expiratory pressure, were therefore not included. Finally, a larger sample size and a longitudinal design would have enabled us to make more detailed inferences and to better understand the effects of ageing on pulmonary functional and structural impairments. The limitations of the sample size warrant caution when interpreting the weight of the results.

## Conclusion

In conclusion, middle-aged people with cervical and upper thoracic SCI many years after injury can have substantial pulmonary functional impairments, whereas structural impairments do not seem to differ considerably from the general population. Further larger and longitudinal studies are needed to determine how pulmonary functional and structural impairments develops over time among people ageing with cervical and upper thoracic SCI, and the clinical impact of these changes. Detailed assessments, such as those used in the present study, are considered valuable in future research and may subsequently be used as tools in the clinical setting.

## Disclaimer statements

**Contributors** None.

**Funding** SPICA is supported by a PhD position grant from the Promobilia Foundation (grant number F18515), and by project grants from the Promobilia Foundation, the Norrbacka-Eugenia Foundation, the Swedish Association for Survivors of Accident and Injury (RTP), Skåne County Council Research and Development Foundation, Skåne University Hospital (SUS Stiftelser och Donationer), Stiftelsen för bistånd åt rörelsehindrade i Skåne, and NEURO Sweden. The main funding body of The Swedish CArdioPulmonary bioImage Study (SCAPIS) is the Swedish Heart-Lung Foundation. The SCAPIS is also funded by the Knut and Alice Wallenberg Foundation, the Swedish Research Council and VINNOVA (Sweden’s Innovation agency), the University of Gothenburg and Sahlgrenska University Hospital, the Karolinska Institute and Stockholm County Council, Linköping University and Linköping University Hospital, Lund University and Skåne University Hospital, Umeå University and Umeå University Hospital, Uppsala University and Uppsala University Hospital.

**Data availability** All the data were archived according to the Swedish Act concerning the Ethical Review of Research Involving Humans to attain confidentiality and are available from the corresponding author upon reasonable request.
